# Understanding the Impact of Chronic Pain in the Emergency Department: Prevalence and Characteristics of Patients Visiting the Emergency Department for Chronic Pain at an Urban Academic Health Sciences Centre

**DOI:** 10.1080/24740527.2019.1587290

**Published:** 2019-05-06

**Authors:** Rebecca N. Small, Yaadwinder Shergill, Steve Tremblay, Jennifer Nelli, Danielle Rice, Catherine Smyth, Patricia A. Poulin

**Affiliations:** a Faculty of Medicine, Memorial University of Newfoundland, St. John’s, Newfoundland, Canada; b The Ottawa Hospital Research Institute, Ottawa, Ontario, Canada; c Centre for Collaborative Health, Oakville, Ontario, Canada; d Department of Anesthesiology and Pain Medicine, University of Ottawa, Ottawa, Ontario, Canada; e Department of Anesthesiology and Pain Medicine, The Ottawa Hospital, Ottawa, Ontario, Canada; f Department of Anesthesiology, Hamilton Health Sciences Centre, Hamilton, Ontario, Canada; g Department of Psychology, McGill University, Montreal, Quebec, Canada; h Department of Psychology, The Ottawa Hospital, Ottawa, Ontario, Canada

**Keywords:** Chronic pain, emergency department, prevalence, opioids, mental health

## Abstract

**Background**: Canadians make approximately 16 million visits to the emergency department (ED) each year. ED visits for non-urgent reasons contribute to suboptimal patient care and ineffective resource use.

**Aims**: To estimate the proportion of ED visits related to chronic pain at our institution. **Methods**. We conducted a retrospective review of 1000 randomly selected ED visits at TOH during the 2012–2013 fiscal year (April 1, 2012 to March 31, 2013). Visits for chronic pain were identified using pre-defined criteria. Demographic and medical data were extracted from medical charts.

**Results**: 104 visits during this time period were related to chronic pain (10.4%; 95% CI: 8.2–12.6). All visits were from unique patients (i.e., no patients contributed more than 1 visit). Patients were predominantly women (71%), with a mean age of 45.9 years. Seventy-eight percent of patients had a primary care provider. The most common location of pain included the abdomen (24%), the head or face (21%), and the low back (21%). Only 5% of patients had consultation with a pain medicine specialist while 78% were awaiting a consultation. More than 2/3 of patients (71%) reported using opioids for their pain.

**Conclusion**: Presenting to the ED for chronic pain was found to occur among a sample of ED visits reviewed. This can result in ineffective care for patients with chronic pain. Cost-effective solutions to improve clinical outcomes and reduce ED use for chronic pain may yield significant improvements in health outcomes of patients and benefits for the health care system.

## RÉSUMÉ

**Contexte**: Les Canadiens font approximativement 16 millions de visites aux urgences chaque année. Les visites aux urgences pour des raisons non urgentes contribuent à ce que les soins aux patients soient sous-optimaux et que les ressources soient utilisées de manière inefficace.

**Objectifs**: Estimer la proportion de visites aux urgences liées à la douleur chronique à notre institution.

**Méthodes**: Nous avons fait un examen rétrospectif de 1 000 visites aux urgences de l’Hôpital d’Ottawa sélectionnées de manière aléatoire qui avaient eu lieu au cours de l’exercice financier 2012-2013 (1^er^ avril 2012 au 31 mars 2013). Les visites en raison d’une douleur chronique ont été repérées à l’aide de critères prédéfinis. Les données démographiques et médicales ont été extraites des dossiers médicaux.

**Résultats**: Au cours de la période à l’étude, 104 visites étaient liées à la douleur chronique (10,4 %; IC 95% : 8,2 – 12,6). Toutes les visites étaient le fait de patients uniques (i.e. aucun patient n’a contribué pour plus d’une visite). Les patients étaient en majeure partie des femmes (71 %), dont l’âge moyen était de 45,9 ans. Soixante-dix huit pour cent des patients avaient un prestataire de soins primaires. L’endroit où la douleur se manifestait le plus fréquemment était l’abdomen (24 %), la tête ou le visage (21 %) et le bas du dos (21 %). Seulement 5 % des patients avaient consulté un médecin spécialiste de la douleur tandis que 78 % étaient en attente d’une consultation. Plus de 2/3 des patients (71 %) ont déclaré avoir utilisé des opioïdes pour leur douleur.

**Conclusion**: Il a été constaté que parmi l’échantillon de visites à l’étude, des patients s’étaient présentés aux urgences en raison d’une douleur chronique. Cette situation peut avoir pour conséquence que les soins prodigués aux patients souffrant de douleur chronique soient inefficaces. La mise en place de solutions efficientes pour améliorer les résultats cliniques et diminuer le recours aux urgences pour de la douleur chronique pourrait grandement améliorer les résultats de santé des patients tout en étant avantageux pour le système de santé.

## Introduction

With Canadians making approximately 16 million visits to the emergency department (ED) each year, ED resources are stretched thin and the opportunity for patients to be managed optimally in the ED is reduced. In a study comparing the health care usage patterns of 11 countries, Canada was reported as having the highest percentage of its population visiting the ED at least once per 2 years (44%), as well as the longest wait times in the ED.^1^ Patients often wait longer to see a physician than is recommended by the Canadian Association of Emergency Physicians guidelines, as determined by their Canadian Emergency Department Triage and Acuity Scale level.^2^ Longer wait times contribute to delayed management of pain or discomfort, patient dissatisfaction with health care services, as well as patients leaving the ED without treatment.^1^ Reducing the number of non-urgent visits to the ED is therefore paramount.

**Figure 1.**
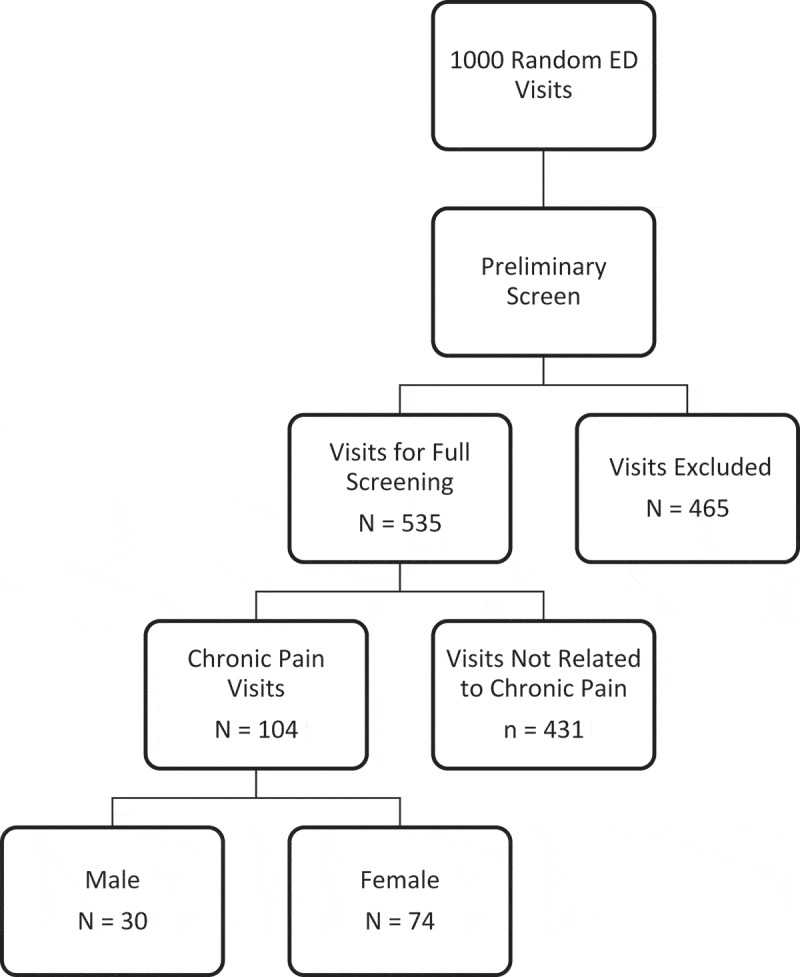
Participant Flow Diagram.

The increased use of the ED for non-urgent medical conditions such as chronic pain has come to the forefront, as ED resources have become increasingly less accessible.3–6 Chronic pain, which affects approximately 20% of the general Canadian population,7,8 accounts for 12–16% of ED visits in the US,9,10 though data discussing the percentage of Canadian ED visits related to chronic pain are difficult to obtain. Some patients with chronic pain rely heavily on the ED as one way to receive treatment, even though it is generally not the appropriate setting to treat chronic pain.^11^ Overreliance on the ED to address chronic pain can result in inappropriate care as well as serious adverse events.8,12,13 Dhalla et al. found that from January 1991 to May 2007, opioid prescriptions in Ontario increased by 29%, while opioid-related deaths doubled from 1991 to 2004 (13.7 to 27.2 per million). Of 3066 patient deaths reviewed, 2037 patients had visited a physician in the 4 weeks preceding their demise and 1807 patients had been to the ED at least once in the year prior. For 378 of the patients reviewed, the ED was their final encounter with the health system, with an average of 11 days between ED visit and death. While it is unclear if chronic pain was a major reason for opioid use in this group, the increase in the prescription of opioids for pain was an important contributor identified in the overall trend observed.^13^

For a variety of reasons, physicians may feel a sense of anger and helplessness when dealing with patients with chronic pain in the ED setting. It can be difficult to differentiate chronic pain sufferers from individuals with a substance use disorder, especially if they are suffering from comorbid physical or mental illness.^8^ Marks and Sachar’s 1973 study, while dated, makes an important point about the assessment of pain by physicians; this study was conducted by 2 psychiatrists who were frequently consulted to assess suspected drug-seeking behavior in patients. They concluded that the behaviour of the majority of these patients could be better explained by their experience of untreated pain.^14^ Several studies have been conducted which have illustrated that many physicians are not well-versed in estimating pain, usually underestimating its severity.^15^ This may relate, in part, to inadequacies in training of chronic pain. O’Rorke et al. surveyed 572 physicians with 76% of respondents reporting that they found chronic pain patients frustrating, and only 34% of respondents reported feeling comfortable managing patients with chronic pain. In this same survey, up to 32% of respondents described their pain education in medical school and residency as “limited,” while 20% said that they received no training at all.^16^

Patients presenting to the ED with chronic pain may present with suboptimal coping skills, severe disease, complex comorbidities and related stress and anxiety,^4^ and the hope for expedient delivery of care and relief of pain.8,17–19 The patient’s expectations coupled with the physician’s expectations and lack of resources can result in poor health outcomes for individuals with chronic pain. The presentation of patients with chronic pain to the ED also suggests that patients may not be receiving adequate resources and support through other avenues such as outpatient or community-based services.^4^

In order to develop a comprehensive and effective strategy to address the issue of chronic pain in the ED, it is important to understand its scope at both the local and regional level, as well as understand the impact of other contributing factors. This may include inefficiencies with the organization of care or access to resources, among others.^20^ Understanding institution level data allows for interventions to be tailored most appropriately to the setting they are being applied in. The objectives of this study were to: 1) estimate the proportion of chronic pain-related visits at The Ottawa Hospital (TOH) ED; and 2) describe the demographic and medical characteristics of patients with CP presenting to the ED.^21^

We expected that the proportion of chronic pain-related visits at TOH ED would be similar to what is reported in the literature; specifically, we expected that approximately 15% of visits would be related to chronic pain. We also expected that fewer patients with chronic pain presenting to the ED would have a primary care provider (PCP) when compared to general population in our region where approximately 91% of Ottawa residents have access to a PCP.22–24

## Material and Methods

Following approval from the Ottawa Health Sciences Network Research Ethics Board, we conducted a retrospective chart review of 1000 randomly selected ED visits at TOH during the 2012–2013 fiscal year (April 1, 2012 to March 31, 2013). The list of randomly selected visits was generated independently by TOH data warehouse from a total of 158,684 ED visits during the period of interest.^25^ TOH is an urban teaching hospital affiliated with the University of Ottawa.

### Inclusion Criteria and Data Collection

We used the International Association for the Study of Pain (IASP) definition of chronic pain as pain that has lasted for longer than three months and that can be either *constant* (e.g. low back pain, fibromyalgia) or *recurrent* (e.g. migraine, nephrolithiasis).^15^ An ED visit was considered to be related to chronic pain if one of the following criteria was met:

1. A chronic pain diagnosis was listed in the “Past Medical History” section of the ED record of the visit or nursing triage document; e.g., fibromyalgia, low back pain, post-herpetic neuralgia^26^ and the symptoms precipitating the visit were congruent with this diagnosis.
2. There was documentation that the patient was taking opioids or co-analgesics for chronic pain; i.e. pregabalin, duloxetine, and/or NSAIDs, and evidence that the pain was present at previous hospital encounters (either in the ED or with a specialist) three months (or more) prior to the ED visit in question.
3. There was documentation from the ED physician or the ED triage nurse stating that the length of time the patient has suffered with pain related to the presenting complaint was at least 3 months.
4. There was evidence of chronic pain in the electronic medical record, determined by reviewing previous encounters/notes from the ED and specialists, that pain had existed for at least 3 months.
5. The electronic medical record had chronic pain noted as the presenting complaint, presenting diagnosis and/or final diagnosis.

### Data Collection

Using the information obtained from the electronic medical records, a clinical research coordinator (YS) with training in chronic pain management completed a preliminary screen of the visits focusing on presenting complaint, presenting diagnosis, and final diagnosis to identify which visits could be related to pain. To ensure the reliability of the initial screen, a pain physician (CS) performed an independent review of 10% of the initial 1000 visits; there was a 95% agreement between the pain physician and clinical research coordinator.

If pain was determined to be present in any of the three fields identified for screening, the case was flagged for further review and data collection of pertinent demographic and medical characteristics was undertaken. This was done in duplicate by two senior anesthesia residents (ST and JN), a chronic pain physician (CS) and a chiropractor (clinical research coordinator) and any discrepancy between two reviewers was resolved during a consensus meeting that involved all 3 reviewers and the clinical research coordinator. The information collected included age, gender, medical conditions, area(s) of pain, psychological problems, and psychosocial challenges that were noted on the documentation related to the visit.

### Statistical Considerations

The data was entered into Statistical Package for Social Sciences (SPSS) software.^27^ Descriptive statistics were calculated and reported for all variables. The proportion of visits related to chronic pain with a 95% confidence interval was calculated. The sample size of 1000 visits was chosen to restrict the total width of a 95% confidence interval around the estimated proportion. In particular, for a sample size of 1000 visits, the two-sided 95% confidence interval around an anticipated proportion of 15% has a total width of 4.4% (margin of error 2.2%).

## Results

Of the 1000 randomly selected ED visits, 465 cases were excluded from full review as they clearly related to an acute condition (Fig. 1). The 535 cases that remained were subsequently reviewed, of which 104 were determined to be related to chronic pain (10.4%; 95% CI: 8.2–12.6). All 104 chronic pain-related visits were from unique patients (i.e., no patients contributed more than 1 visit). Patients in this group were predominantly women (71%) and the mean age was 45.9 (standard deviation [SD] = 18.7) years, with an age range of 18 to 90 years. The majority of patients (78%) had a PCP. There was no missing data in the sample, although there could have been incomplete documentation within a patient’s chart.

The patient population that presented to the ED with chronic pain presented with multiple diverse comorbidities. The most common chronic pain locations were the abdomen (24%), the head or face (21%), and the low back (21%). The other locations of chronic pain were thoracic (9%), lower limb (8%), perineal/genital/anal (6%), shoulder/upper arm (3%) and pelvic (2%). Six percent of patients reported multiple regions of pain. Medical comorbidities were common with 80% of patients having at least one comorbid condition that was not related to their reason for admission. The most prevalent conditions were hypertension (23%), arthritis (15%) and diabetes (14%). More than 2/3 of patients (71%) reported using opioids for their pain.

**Figure 2.**
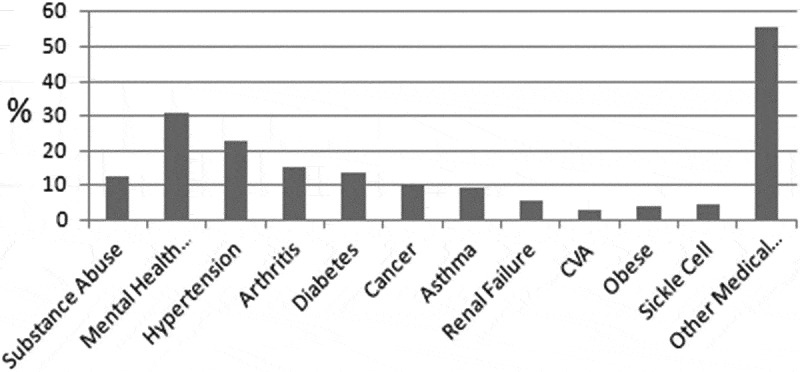
Predominant medical and mental health comorbidities documented among patients with chronic pain.

Mental health comorbidities were also common (see Fig. 2), with 31% of the patients with chronic pain having a documented mental health condition, with the most prevalent being depression (19%), followed by anxiety (13%), substance use disorder (13%), and with 14% of patients having more than one mental health condition. According to the electronic medical record documentation available, only 12% of patients reviewed previously had an appointment with a mental health specialist at the hospital.

The majority of patients had been assessed by multiple specialists including: pain medicine (78%), neurology (23%), gastroenterology (22%), general surgery (19%) and cardiology (14%), however, just 5% of patients had previously been assessed by a pain medicine specialist at the institution where this study took place.

## Discussion

Although the proportion of ED visits attributed to chronic pain among a sample of patients in our institution was slightly below the hypothesized prevalence, our results are in line with data obtained from previous studies, suggesting that chronic pain accounts for 10–16% of all visits to the ED.9,28–30 This represents approximately 18,000 ED visits per year at TOH. The patient population with chronic pain that presented to TOH ED reported similar rates of abdominal pain (24%), head/face pain (21%) and lumbar pain (21%) as seen in previous studies.9,30,31 The majority of our patients (79%) did have access to a PCP, which is consistent with findings by Blank et al. (85–93%).^32^

It is important to consider what contributes to patients visiting the ED rather than their PCP. A study published in 2017 by MacKichan et al. found that past difficulties in access to primary care had a large influence on whether or not a patient chooses to present to the ED. One factor that was identified as problematic was difficulty in navigating complex and ever-changing appointment booking systems, causing frustration for both patients and staff while fostering mistrust in the system. A lack of available primary care appointments has been quoted as another factor driving patients toward the ED.^33^ The perceived lack of urgent primary care appointments was verified in a qualitative study of patients with repeated visits to the ED for chronic pain. Ansell et al. conducted a systematic review identifying primary care wait times as a growing problem for the Canadian population. Patients are often unable to schedule same-day appointments with their PCP, resulting in increased presentation to urgent care for non-urgent health concerns. Ansell et al. found that several interventions were effective in improving availability of primary care appointments including open access scheduling (increasing access to same-day appointments), incorporation of nurse practitioners into medical practice, providing telephone calls for follow-up consultations and the establishment of PCPs practicing in health teams.^34^

PCPs and ED physicians alike may struggle to address the issue of chronic pain in their patients as physicians receive little to no formal training on chronic pain management. In 2009, Watt-Watson et al. surveyed 10 major Canadian universities that had a medical and nursing program and found that 68% of sites had no time allotted specifically to chronic pain management in their curriculum, though several programs claimed they were unable to give specific numbers due to the integrated nature of their programs. The mean number of hours spent on CP management in medicine was 16. Many health programs noted that chronic pain management was mainly addressed during clinical placements, thus experience varied greatly between students.^35^ Acquiring specialized knowledge through chronic pain courses offered in medical and anesthesia faculties are one recommendation that may help to provide important information about pain management in the ED. Project ECHO (Extension for Community Healthcare Outcomes) Chronic Pain and Opioid Sterwardship is another interesting model of continuing medical education that can help bridge knowledge, self-confidence, and competency gaps in chronic pain management.^36^

To supplement training about chronic pain management for ED physicians, a priority-setting process led to the identification of key elements to incorporate in online modules.^37^ Examples of learning objectives of online modules include: educating the patients of risks of different classes of pain medications and understanding in-hospital resources available for patient follow up and referral to pain clinics. Implementing and evaluating the impact of providing additional training for managing patients with chronic pain is an important next step in determining ways to improve the care of patients arriving at the ED for chronic pain.

Physicians may also have significant problems accessing expert opinions when it comes to the management of chronic pain in their patients. Peng et al. surveyed multidisciplinary pain treatment facilities (MPTF) in Canada to explore the services provided by these facilities, as well as the access to care for Canadians with chronic pain. They found that most MPTFs (80%) were concentrated in large cities, and no services were available in the province of Prince Edward Island or the Canadian territories. On average, one MPTF was available for every 258,000 Canadians. Wait times were highly variable, which was mainly dependent on the principal type of funding the MPTF received (public or non-public), with public facilities having an average wait time of 6 months, while non-public facilities had an average wait time of only 2 weeks.^38^

Time and resources in the ED are limited, and often do not result in optimal recognition and treatment of a chronic pain syndrome in the ED.^39^ However, given that the ED is often a last resort for patients who present with chronic pain, the ED should provide supportive and integrated practices for chronic pain syndromes again underscoring the importance of further training for ED physicians and staff.

The high proportion of patients with chronic pain that presented with a history of mental health conditions (30%) – which is likely to be underestimated given that ED records do not systematically contain this information – highlights the fact that a biopsychosocial approach is required to appropriately address all need of the patients experiencing chronic pain. Choi et al.^40^ reported that depression and chronic pain independently predict ED visits in low-income adults aged 50 years and older. Also, Woodhouse et al. outlined that an ED–based behavioural health consultation may be useful in reducing the high utilization of ED services by some patients with chronic pain.^41^ More recently, Rash et al.^42^ found that an inter-disciplinary chronic pain management program for high frequency users of the ED was effective in improving clinical outcomes and reducing acute care utilization.^42^ Therefore, being able to identify the proportion of patients with chronic pain with comorbid mental health and behavioural problems will further help to delineate the type and amount of resources (psychiatry, psychology, social work, nurse practitioner) needed to reduce the barriers to appropriate chronic pain patient care in and out of the ED and to potentially reduce and avoid future admissions.

Finally, more than two-thirds of the patients in this study were taking opioids for their pain, despite the evidence that their beneficial effects are small and likely comparable to non-opioid alternatives^43^ and that their use is associated with some degree of risks including dependence,^44^ overdoses and deaths.^13^ We were unable to ascertain how long patients were on opioids or dosage, but the fact that they presented to the ED for their pain and were already prescribed opioids indicates that a health professional was aware of their condition, that they were receiving treatment but that this was insufficient. In a separate study where we surveyed and interviewed 59 patients who presented to the ED for their pain, inability to cope with pain was one of the most common reason cited by patients for presenting to the ED,^29^ again reinforcing the importance of a multipronged approach that provides patients with a wide array of strategies to manage their main. Several studies have demonstrated the benefits of non-pharmacological interventions such as pain education,^45^ physical therapy,^46^ cognitive behavior therapy,^47^ and mindfulness-based interventions,^48^ among many others. These are good options to discuss with patients in the ED for more comprehensive management.

There are limitations to this study; we only examined chronic pain prevalence over a 1-year period and at one urban center among a random sample of ED visits. The proportion of chronic pain-related presentation in rural, remote or underserviced areas where access to even basic medical care in the community is tenuous, but this remains to be tested empirically. An additional consideration when interpreting findings of this study is the inherent limitations associated with retrospective chart review study designs. We were reliant on what was recorded in the electronic medical record and aspects such as psychosocial comorbidities are often underreported in patient records. This study designed also did not allow us to consider more finite details of presentation to the ED such as time of presentation and how this may influence results, especially if patients attended the ED at times when their PCP would have been closed. The results should be interpreted with this understanding and future research which prospectively collects data and these additional variables is necessary.

## Conclusion

We have estimated the prevalence of patients with chronic pain that are present in the ED at our institution, and found similar results to those in other institutions in North America. This fact is often overlooked because there is generally no unique billing code for chronic pain, making chronic pain difficult to capture using health system level data. Determining how best to reduce chronic pain-related ED visits may allow for a better understanding of how outpatient and community services can meet the needs of patients with chronic pain. This would positively influence ED’s by reducing overcrowding and the cost of acute care resources devoted to non-urgent problems and importantly, this could improve health outcomes for individuals with chronic pain.

We have also asserted that patients with chronic pain presenting to the ED often have complex difficulties, likely requiring a multidisciplinary approach that targets the biopsychosocial factors contributing to chronic pain. This is best accomplished through non-emergent medical care. However, we acknowledge that chronic pain problems, especially acute exacerbations of chronic pain syndromes, will likely continue to be common presenting complaints in the ED. Patients facing a pain crisis who feel at a loss of how to manage flare-ups need support and treatment, and the ED is an important actor in this regard. Therefore, training ED physicians in various approaches to the management of chronic pain and connecting EDs with pain management programs to ensure appropriate follow-up care are important steps to improve the overall care of patients with chronic pain.

## Data Availability

Data is available on request from the corresponding author.
